# Outcomes Associated with Mitral Regurgitation Reduction and Myocardial Work After Transcatheter Edge-to-Edge Repair of a Mitral Valve in Dogs

**DOI:** 10.3390/vetsci13060597

**Published:** 2026-06-19

**Authors:** Soontaree Petchdee, Xufeng Ying, Suchada Huttayananont, Kotchapol Jaturanratsamee, Chattida Panprom, Wannisa Meepoo, Ratikorn Bootcha

**Affiliations:** 1 Department of Large Animal and Wildlife Clinical Science, Faculty of Veterinary Medicine, Kasetsart University, Kamphaeng Saen, Nakorn Pathom 73140, Thailand; 2 Science and Innovation for Animal Health Program, Graduate School, Faculty of Veterinary Medicine, Kasetsart University, Bangkok 10900, Thailand; 3 Kasetsart University Veterinary Teaching Hospital, Faculty of Veterinary Medicine, Kasetsart University, Kamphaeng Saen, Nakorn Pathom 73140, Thailand

**Keywords:** MMVD, mitral regurgitation, myocardial work, speckle-tracking echocardiography, TEER

## Abstract

Transcatheter edge-to-edge repair (TEER) is a recent minimally invasive method of managing mitral regurgitation (MR) in dogs with myxomatous mitral valve disease (MMVD). As the goal of intervention is to minimize MR severity, this study aimed to determine the association between reduced MR and changes in myocardial work indices after TEER in dogs. Ten client-owned dogs with moderate-to-severe MR were enrolled in the study, and all underwent TEER with multimodal imaging guidance. Myocardial work was analyzed before and after the procedure, and the MR severity, transmitral pressure gradients, left atrial and ventricular measurements, and index of myocardial work (GWI: the total myocardial work during systole; GCW: work contributing to LV ejection; GWW: ineffective work that contributes to no forward displacement; and GWE: ratio of constructive work to total work) were calculated. TEER significantly reduced MR severity in the majority of dogs, and this MR decrease was associated with a greater efficiency of myocardial work, more constructive work, and less wasted energy. No significant negative associations of moderate post-procedure gradients with short-term clinical outcomes emerged. TEER-mediated reduction in MR improves myocardial function in dogs. However, long-term studies are also needed to examine the effects of residual MR and transmitral gradients on cardiac function and clinical outcome.

## Simple Summary

In this study, we evaluated the effect of transcatheter edge-to-edge repair (TEER), which is a minimally invasive modality for mitral regurgitation (MR) in dogs with myxomatous mitral valve disease (MMVD). Ten dogs with moderate-to-severe MR received TEER under imaging guidance. The intervention effectively decreased MR severity in the majority of dogs and maximized myocardial work efficiency with greater constructive cardiac work and a decreased amount of wasted energy. These findings suggest that TEER can improve cardiac function in dogs with MMVD. However, longer-term studies are required to assess its benefit relative to its absence and its impact on residual MR.

## 1. Introduction

Myxomatous mitral valve disease (MMVD) is a known and prevalent cause of mitral regurgitation (MR) in dogs and an important cause of morbidity and mortality in small animal cardiology [1,2,3]. Gradual degeneration of the valve leads to chronic volume overload, enlargement of the left atrium, and finally, congestive heart failure [4,5,6]. Current medical management of MMVD primarily includes pimobendan, loop diuretics, angiotensin-converting enzyme inhibitors, and aldosterone antagonists, which improve clinical signs and prolong survival in affected dogs. However, these pharmacological therapies do not directly address the structural abnormalities of the mitral valve responsible for regurgitation. In the field of veterinary medicine, transcatheter edge-to-edge repair (TEER), a less invasive method for mitral valve repair than open-heart surgery, has recently been developed, and it has been applied to the mitral valve leaflets to decrease regurgitant flow and avoid the need for cardiopulmonary bypass [7,8,9,10].

The TEER procedure has the trade-off of decreasing the effective mitral valve orifice area and increasing transmitral pressure gradients. Optimizing these conflicting effects will be key to the success of the procedure [11,12,13]. In addition to conventional echocardiographic parameters, myocardial work analysis based on strain echocardiography has been identified as a new tool for evaluating cardiac function [14,15,16,17]. Unlike conventional indices, myocardial work combines myocardial deformation and afterload, offering a more complete indication of ventricular function in conditions such as MR, in which there are changes in the work loading. The relative extent of MR reduction to changes in myocardial work in dogs receiving TEER is poorly defined [18]. Moreover, the clinical relevance of post-procedural transmitral gradients has been established to a limited extent in veterinary practice, particularly in those with residual MR. The knowledge gained from examining the interaction of these processes may enhance patient selection, intervention planning, and outcome prediction.

The objective of this study was to determine whether reductions in MR and myocardial work indices after TEER in dogs with MMVD were associated with hemodynamic and functional consequences of residual MR and post-procedural transmitral gradients. TEER was performed as an early structural intervention aimed at reducing regurgitant volume and delaying progression to congestive heart failure. We hypothesized that successful reduction of mitral regurgitation following TEER would be associated with improvements in myocardial work indices, reductions in cardiac chamber remodeling, and favorable hemodynamic outcomes without clinically significant increases in transmitral pressure gradients.

## 2. Materials and Methods

### 2.1. Study Design and Ethical Approval

This prospective observational study involves client-owned dogs diagnosed with myxomatous mitral valve disease (MMVD) and moderate-to-severe mitral regurgitation (MR). Dogs were recruited from Kasetsart University Veterinary Teaching Hospital, Kamphaeng Saen campus, cardiology service. Inclusion criteria included (1) echocardiographic evidence of MMVD, with at least moderate MR; disease severity was classified according to the American College of Veterinary Internal Medicine (ACVIM) consensus guidelines for MMVD. Dogs enrolled in the study included ACVIM Stage B2, C, and D patients. (2) body weight and anterior–posterior mitral valve leaflet diameter (AP) access compatible with TEER intervention; and (3) clinical signs and risk for the TEER procedure. Dogs were excluded if they had (1) an anterior–posterior mitral leaflet diameter < 12 mm, (2) clinically relevant mitral stenosis, (3) congenital heart disease, (4) severe systolic dysfunction unrelated to volume overload, (5) severe pulmonary hypertension, or (6) any systemic illness deemed likely to interfere with anesthesia or follow-up. The general characteristics of dogs in this study are presented in Table 1. All procedures were performed with the owners’ informed consent, and the study protocol was approved in accordance with the Kasetsart University animal care and use committee (ACKU68-VET-086). The sample size calculation was not performed in the current study, as a pilot observational study including all eligible dogs presented during the study period and TEER is a novel procedure in veterinary medicine, and case availability was limited.

### 2.2. Pre-Procedural Assessment

All dogs underwent a standardized physical examination; thoracic radiographs were obtained in two standard views, including right lateral and ventrodorsal; a standard six-lead electrocardiogram (leads I, II, III, aVR, aVL, and aVF) was recorded in all dogs before the procedure; and full transthoracic (TTE) and transesophageal (TEE) echocardiography prior to intervention, as previously described [19,20]. Echocardiographic examinations were performed using a Vivid E95 ultrasound system (GE Healthcare, Horten, Norway) equipped with M5Sc-D (1.5–4.6 MHz) transthoracic and 9VT-D transesophageal transducers. Image acquisition and analysis were performed using EchoPAC software version 204 (GE Healthcare, Horten, Norway). The left atrial-to-aortic root ratio (LA/Ao), left ventricular internal dimensions in diastole and systole (LVIDd, LVIDs), and fractional shortening were measured; moreover, MR severity was determined semi-quantitatively via color Doppler assessment of the jet area and other supportive indices, e.g., vena contracta width and regurgitation volume. The transmitral pressure gradients at baseline were determined by continuous-wave Doppler. Dogs with evidence of elevated baseline gradients suggestive of functional mitral stenosis were excluded from the study.

### 2.3. Transcatheter Edge-to-Edge Repair

All procedures were performed under general anesthesia with continuous hemodynamic and electrocardiographic monitoring. Dogs were premedicated with midazolam (Dormicum^®^, 5 mg/mL, Roche, Basel, Switzerland) at 0.2 mg/kg intravenously. Anesthesia was induced with propofol (Propofol-Lipuro^®^, 10 mg/mL, Melsungen, Germany) at 2–6 mg/kg IV to effect and maintained with isoflurane (Forane^®^, Abbott Laboratories, Chicago, IL, USA) in 100% oxygen following endotracheal intubation. End-tidal isoflurane concentration was maintained between 1.2% and 2.0% according to anesthetic requirements. Intraoperative analgesia was provided with fentanyl (Fentanyl-Hameln^®^, 50 μg/mL, Hameln, Germany) administered as a continuous rate infusion at 5–10 μg/kg/h. Mechanical ventilation was adjusted to maintain end-tidal carbon dioxide between 35 and 45 mmHg. Continuous perioperative monitoring was performed using a multiparameter monitor (BeneVision N22, Mindray Medical International Ltd., Shenzhen, China) and included electrocardiography, pulse oximetry, capnography, invasive arterial blood pressure monitoring via dorsal pedal artery catheterization, central venous pressure, and body temperature. Arterial blood gas analysis was performed using an i15 Vet Blood Gas and Chemistry Analyzer (EDAN Instruments, Inc., Shenzhen, China). Lactated Ringer’s solution (Lactec^®^, Otsuka Pharmaceutical Co., Ltd., Tokyo, Japan) was administered intravenously at 3–5 mL/kg/h and adjusted according to invasive hemodynamic measurements. Dobutamine (Dobutrex^®^, 12.5 mg/mL, Eli Lilly and Company, Indianapolis, IN, USA) was administered as a continuous rate infusion at 5–20 μg/kg/min when required to maintain cardiovascular stability.

A transapical puncture was performed to access the left atrium with transesophageal echocardiographic guidance after an introducer sheath was placed. A delivery system was deployed into the left atrium, and the mitral valve was visualized by TEE, as in a previous study [21,22]. Device orientation and positioning were optimized to best fit with the regurgitant jet. The grasp of the leaflets was performed by moving the delivery system across the mitral valve into the left ventricle and subsequently retracting the clip from both anterior and posterior mitral leaflets.

The appropriate leaflet insertion and reduction in MR were confirmed in real time by TEE imaging. Procedural success was defined as successful implantation of the device with a reduced MR severity of at least one grade (≥1) with no major intraoperative complications. Patients were monitored after treatments for hemodynamic stability and arrhythmias during post-procedure recovery. Post-procedural echocardiography was performed 24–48 h after the procedure, with a focus on assessing MR severity, transmitral gradients, and device placement. Follow-up evaluations were conducted at monthly intervals, comprising clinical assessment and echocardiographic inspection, with the attending clinician deciding to adjust the medical therapy based on clinical status.

### 2.4. Echocardiographic and Hemodynamic Analysis

The examinations were performed using a Vivid E95 ultrasound system (GE Healthcare, Horten, Norway) equipped with a 4–6 MHz phased-array transducer and 9VT-D, 3D TEE. Standard echocardiography was performed as previously described in published veterinary practice guidelines [20,23]. MR severity was reassessed after the procedure, following its course, in the same manner as at baseline, to maintain comparability. Transmitral pressure gradients were determined with continuous-wave Doppler from the apical view, and the mean gradients were computed using multiple cardiac cycles, concentrating on detecting high gradients that can signal functional mitral stenosis after TEER.

### 2.5. Severity Assessment of Mitral Regurgitation

The severity of mitral regurgitation was determined using semi-quantitative echocardiographic scores optimized for canine cases, adapted from a previous study [17,24]. Assessment was performed using transthoracic echocardiography with simultaneous two-dimensional and color Doppler imaging. All examinations were performed by an experienced veterinary cardiologist with standardized imaging planes. The MR grading system included two echocardiographic variables: (1) vena contracta (VC) width and (2) mitral valve regurgitant volume (MRV). Measurements were performed on optimized left apical views during systole for at least 3 consecutive cardiac cycles, and the average value was used for analysis. The vena contracta was defined as the narrowest part of the regurgitant jet immediately distal to the mitral valve orifice. VC width was measured in millimeters using color Doppler zoom imaging with Nyquist limits adjusted to minimize aliasing artifacts.

Mitral regurgitant volume was calculated through a Doppler-derived volumetric assessment and is presented in milliliters per beat. Quantification was performed using the standard echocardiographic methodology, calculating the forward and total left ventricular stroke volumes. The scores for each parameter range from 1 to 10 (Table 2). Total MR severity was classified using the combined score from VC width and mitral regurgitant volume. MR severity categories were defined as follows: mild MR (total score: <6), moderate MR (total score: 6 to <12), and severe MR (total score: ≥12). This scoring system was applied before and after TEER to evaluate procedural effectiveness and residual MR severity. The composite MR severity score used in this study should be considered an exploratory semi-quantitative assessment tool derived from established echocardiographic variables rather than a formally validated grading system.

### 2.6. Myocardial Work Analysis

Speckle-tracking and myocardial work analyses were performed offline using EchoPAC software version 204 (GE Healthcare, Horten, Norway), as previously described [25]. Standard apical views (four-chamber, two-chamber, and long-axis) were acquired at high frame rates (usually >60 frames per second) and retained for offline analysis. Global longitudinal strain (GLS) was calculated as the total myocardial strain during the entire cardiac cycle. Non-invasive left ventricular pressure curves were estimated by combining non-invasive systolic blood pressure measurements with the timing of valvular events obtained through echocardiography-based time frames. Pressure-strain loops were built per cardiac cycle, which allowed myocardial work parameters to be estimated, including GWI: the total myocardial work during systole; GCW: work contributing to LV ejection; GWW: ineffective work that contributes to no forward displacement; and GWE: ratio of constructive work to total work. All echocardiographic, strain, and myocardial work measurements were performed by a single experienced veterinary cardiologist who was blinded to clinical outcomes during offline analysis to reduce concerns regarding interobserver variability.

### 2.7. Outcome Measurement

The main outcome of interest was the association between a decrease in MR severity and the change in myocardial work indices post TEER. Concomitant secondary outcomes were alterations in cardiac dimensions, transmitral pressure gradients, and short-term clinical status. Dogs were further stratified by MR and post-procedural transmitral gradients to determine their associations with myocardial work and clinical response (Table 3).

### 2.8. Statistical Analysis

The continuous variables were examined for normality using the Shapiro–Wilk test. Data are shown as mean ± standard deviation. Pre- and post-procedural values were compared using the paired *t*-test, and Pearson correlation analysis was used for normally distributed variables. Group comparisons were made using independent *t*-tests or, where appropriate, Mann–Whitney U tests. Statistical analyses were conducted using commercially available software, with a *p*-value < 0.05 considered statistically significant. All statistical analyses were performed using GraphPad Prism version 10.2.3 (GraphPad Software LLC, San Diego, CA, USA).

### 2.9. Use of Artificial Intelligence (AI) Tools

During manuscript preparation, the authors used the artificial intelligence-based language model ChatGPT (OpenAI, San Francisco, CA, USA; GPT-5.5 model). to assist with language editing, grammatical revision, sentence restructuring, and reference formatting. The AI tool was used only to improve the readability and organization of the manuscript text and was not involved in study design, data acquisition, echocardiographic analysis, statistical analysis, interpretation of results, or generation of scientific conclusions. All AI-assisted outputs were carefully reviewed, revised, and verified by the authors. The authors take full responsibility for the accuracy, integrity, and final content of the manuscript.

## 3. Results

### 3.1. Procedures and Mitral Regurgitation Amelioration

**Figure 1:**
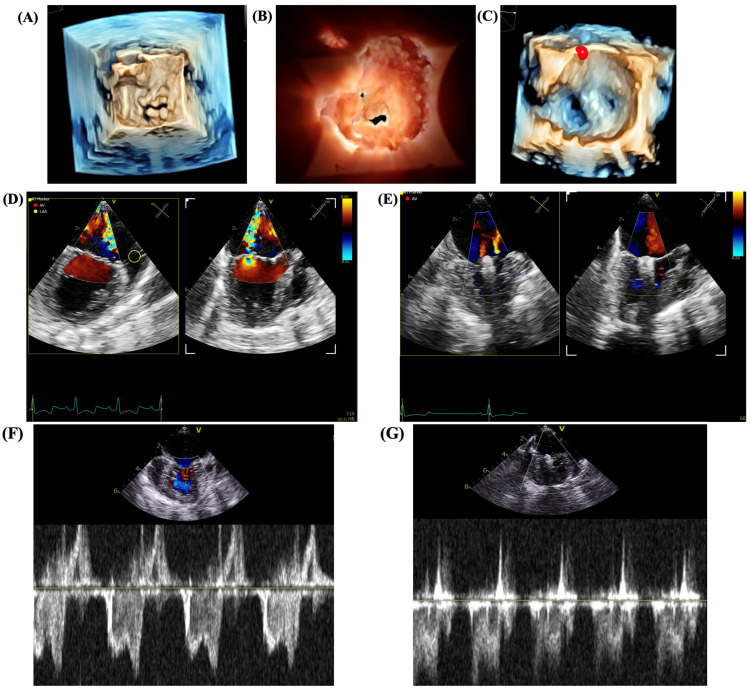
Three-dimensional echocardiographic and Doppler assessment before and after transcatheter edge-to-edge repair (TEER) in dogs with MMVD. (**A**) Three-dimensional transesophageal echocardiographic (3D-TEE) image of the mitral valve before TEER, demonstrating severe leaflet prolapse and incomplete coaptation. (**B**) Intraoperative surgical view of the mitral valve during TEER device implantation. (**C**) Three-dimensional echocardiographic image after TEER, showing successful leaflet approximation and improved coaptation. (**D**) Color Doppler echocardiography before TEER, demonstrating severe mitral regurgitation with a large eccentric regurgitant jet. (**E**) Color Doppler echocardiography immediately after TEER, showing a marked reduction in regurgitant jet area. (**F**) Continuous-wave Doppler assessment of transmitral inflow before TEER, demonstrating a low baseline transmitral pressure gradient. (**G**) Assessment after TEER.

Pre- and post-TEER visualization of the mitral valve anatomy was achieved via three-dimensional echocardiography (Figure 1). Pre-procedural imaging revealed mitral valve coaptation in the form of a large central regurgitant orifice and severe mitral regurgitation (MR). Real-time 3D guidance allowed precise adjustment and grasp of mitral valve leaflets during clip application. Device insertion resulted in a notable decrease in the regurgitant orifice area and improvement in leaflet coaptation. Color Doppler echocardiography demonstrated a significant reduction in MR severity immediately after TEER, and the majority of dogs showed only mild residual regurgitant flow.

In continuous-wave Doppler, the transmitral inflow showed a mild increase in transmitral pressure gradient following TEER implantation (Figure 1F,G). Prior to TEER, the Doppler recording showed the typical mitral inflow due to severe mitral regurgitation and unrestricted transmitral flow (Figure 1F). After TEER, the profile of the transmitral flow with a slight increase in the diastolic inflow velocity and mean transmitral pressure gradient exhibited higher values compared to the control test data and reflected a reduction in the effective mitral valve orifice area after leaflet approximation (Figure 1G). Despite a greater transmitral gradient, no dogs had echocardiographic evidence of clinically significant mitral stenosis during the follow-up. Mean post-procedural transmitral gradients remained within clinically accepted limits, with no evidence of progressive pulmonary edema, severe left atrial hypertension, or clinical deterioration suggestive of increased transmitral flow resistance. Results suggest that TEER effectively decreased MR and maintained sufficient transmitral diastolic flow. The increase in transmitral pressure gradient after TEER was accompanied by a substantial decrease in mitral regurgitation severity and an improvement in measures of myocardial work indices.

TEER decreased multiple echocardiographic parameters (including MR severity) (Table 3). Mitral regurgitation severity score, vena contracta width, and regurgitant volume decreased significantly immediately after intervention and remained improved at the 2-month follow-up. Significant reductions in left atrial and left ventricular dimensions were also observed at follow-up. After TEER, myocardial work analysis showed improvement. Global work index, global constructive work, and global work efficiency improved after intervention, while global wasted work decreased. Although global longitudinal strain became less negative after TEER, myocardial work indices still indicated improved mechanical efficiency in the ventricles following reduction in chronic volume overload. Transmitral pressure gradients were elevated slightly after clip implantation but remained within the clinically acceptable range in the majority of dogs.

### 3.2. Changes in Myocardial Strain and Myocardial Work Following TEER

**Figure 2:**
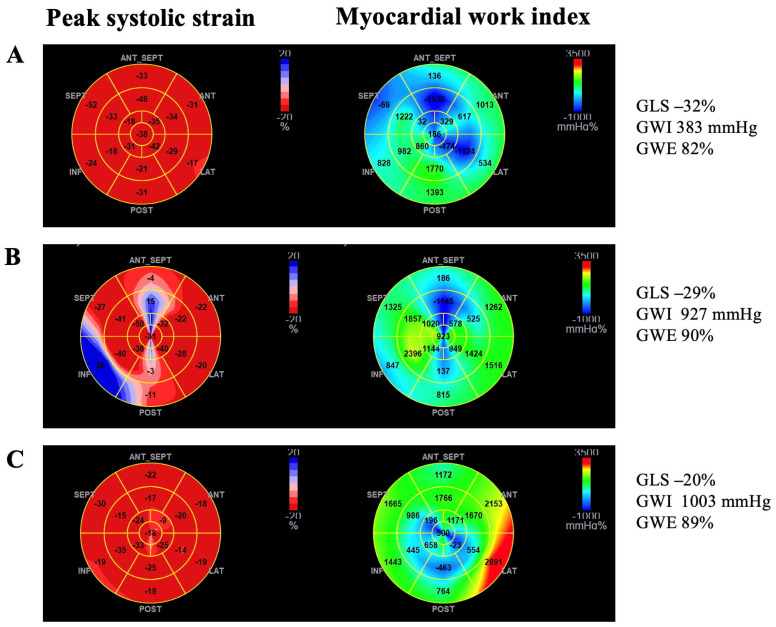
Left ventricular strain and myocardial work after TEER in dogs with MMVD. The bull’s-eye maps of peak systolic strain and myocardial work index before and after TEER. (**A**) Pre-TEER examination of global longitudinal strain (GLS), global work index (GWI), and global work efficiency (GWE). (**B**) Immediate post-TEER examination. (**C**) Two-month follow-up examination.

Figure 2 presents myocardial strain and myocardial work analyses. Prior to TEER (Figure 2A), dogs with severe MR exhibited impaired myocardial work performance, with a reduced global work index (GWI) and global work efficiency (GWE), even though global longitudinal strain (GLS) was preserved or accentuated. The myocardial region showed a heterogeneous regional work distribution by pressure–strain loop analysis, with areas of inefficient contraction of the myocardium, especially at the puncture site in the apical area. After TEER (Figure 2B), myocardial work parameters demonstrated improvement. GWI was elevated (921.7 ± 168 mmHg% prior to intervention to 1149.3 ± 174 mmHg% immediately post-procedure), and GWE improved from 82% to 90%. Despite a marginal decrease in absolute magnitude (−32% to −29%) observed in the GLS, myocardial working efficiency increased, confirming that ventricular mechanical function improved after volume overload decreased (Figure 2C). At a 2-month follow-up assessment, myocardial work parameters improved relative to baseline. GWI rose to 1003 mmHg%, whilst GWE remained unchanged at 89%. At −20%, this reduction in GLS signifies the normalization of loading parameters following a successful MR reduction rather than deterioration in systolic function. We observed a significant flattening of the regional myocardial work distribution at follow-up, suggesting improved ventricular mechanical coordination after TEER.

**Figure 3:**
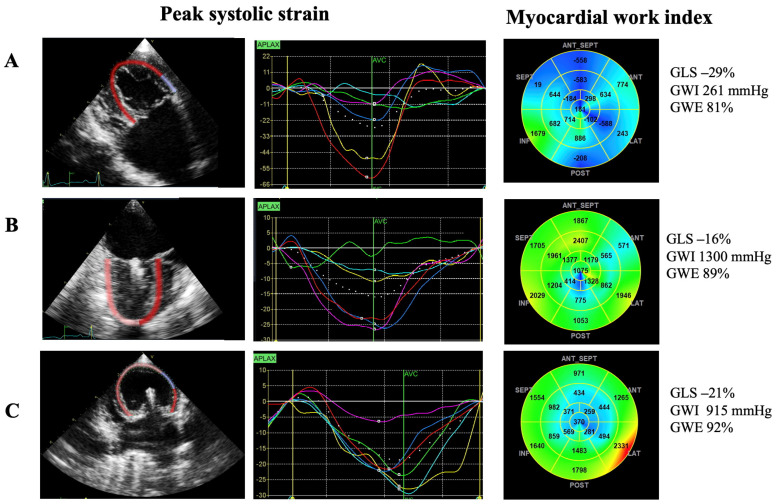
Representative speckle-tracking strain and myocardial work analysis before and after transcatheter edge-to-edge repair (TEER) in a dog. (**A**) Pre-TEER examination of global longitudinal strain (GLS), global work index (GWI), and global work efficiency (GWE). (**B**) Immediate post-TEER examination. (**C**) Two-month follow-up examination.

Figure 3 shows the strain and myocardial work analyses obtained before TEER, immediately after TEER, and at the 2-month follow-up. Prior to intervention (Figure 3A), severe mitral regurgitation was associated with relatively preserved myocardial deformation (GLS = −29%) but reduced myocardial work performance, as evidenced by a low GWI (261 mmHg%) and reduced GWE (81%). Segmental strain curves demonstrated considerable heterogeneity in myocardial deformation, while myocardial work mapping revealed regional areas of reduced constructive work and increased mechanical inefficiency. Immediately after TEER (Figure 3B), successful reduction of mitral regurgitation resulted in substantial improvement in myocardial work indices. Although GLS became less negative (−16%), GWI increased markedly from 261 to 1300 mmHg%, and GWE improved from 81% to 89%. Regional myocardial work distribution became more homogeneous, suggesting improved ventricular mechanical coordination and efficiency following correction of the regurgitant lesion. At the 2-month follow-up (Figure 3C), myocardial work parameters remained improved compared with baseline. GLS partially recovered to −21%, while GWI and GWE remained elevated at 915 mmHg% and 92%, respectively. The bull’s eye myocardial work map demonstrated persistent normalization of regional work distribution with fewer areas of reduced constructive work. These findings suggest that reduction of mitral regurgitation by TEER may lead to favorable ventricular mechanical adaptation and sustained improvement in myocardial work efficiency despite alterations in conventional strain measurements.

Overall, myocardial work analysis provided additional functional information beyond GLS alone, demonstrating that improvements in ventricular efficiency can occur even when strain values become less negative following reduction of chronic volume overload. This pattern is consistent with altered loading conditions and improved myocardial energy after successful TEER.

### 3.3. Correlation Analysis

**Figure 4:**
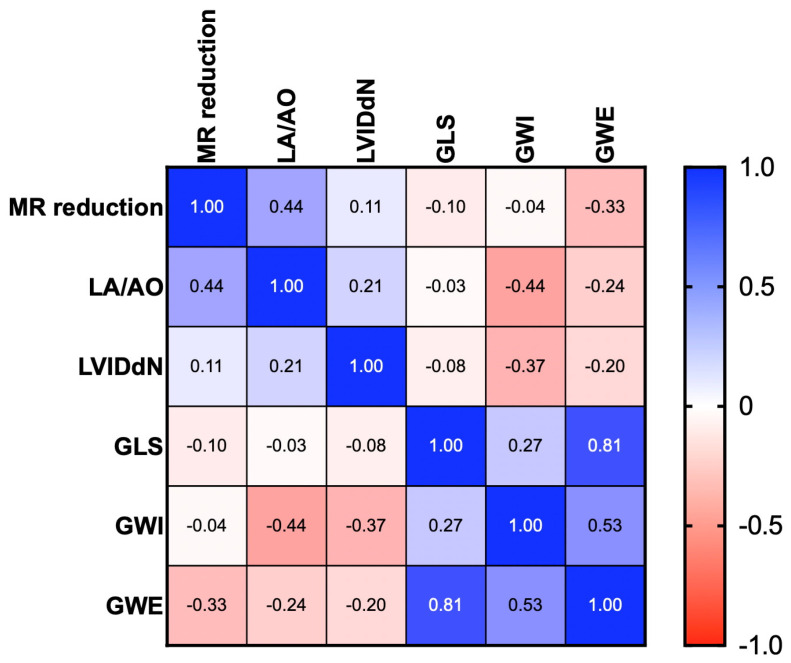
Correlation matrix between mitral regurgitation reduction, echocardiographic remodeling, and myocardial work indices after TEER. Heatmap demonstrating correlation coefficients among mitral regurgitation (MR) reduction, left atrial-to-aortic ratio (LA/Ao), normalized left ventricular internal diameter in diastole (LVIDdN), global longitudinal strain (GLS), global work index (GWI), and global work efficiency (GWE). Positive correlations are represented in blue and negative correlations in red.

Figure 4 illustrates the correlations among mitral regurgitation (MR) reduction, echocardiographic remodeling variables, and myocardial work indices. Global longitudinal strain (GLS) was strongly and positively correlated with global work efficiency (GWE) (r = 0.81, *p* = 0.001), indicating that improved myocardial deformation was associated with enhanced ventricular work efficiency following TEER. A moderate positive correlation was observed between global work index (GWI) and GWE (r = 0.53, *p* = 0.06), although this relationship did not reach statistical significance.

Weak correlations were identified between MR reduction and myocardial work parameters, including the GWI (r = −0.04, *p* = 0.89) and GWE (r = −0.33, *p* = 0.28). Left atrial size (LA/Ao) demonstrated weak negative correlations with GWI (r = −0.44, *p* = 0.13) and GWE (r = −0.24, *p* = 0.43). Similarly, normalized left ventricular internal diameter in diastole (LVIDdN) was negatively correlated with GWI (r = −0.37, *p* = 0.22) and GWE (r = −0.20, *p* = 0.52). Although most correlations did not reach statistical significance, likely due to the small sample size, myocardial work analysis demonstrated a trend toward improved ventricular performance following successful MR reduction. These findings suggest that myocardial work parameters may provide complementary information regarding functional recovery after TEER beyond conventional structural remodeling indices.

## 4. Discussion

In this study, we aimed to evaluate the effect of myocardial work following transcatheter edge-to-edge repair (TEER) in dogs with MMVD and significant MR. The number of dogs for baseline TEER cohort (n = 10), immediate post-TEER analysis of dogs (n = 10), two-month follow-up analysis dogs (n = 6), and correlation analysis dogs (n = 6). The major finding was that TEER decreased MR severity in the majority of dogs and improved the global work index (GWI) and global work efficiency (GWE). These observations indicate that an analysis of myocardial work might be useful in informing ventricular function following mitral valve procedures in veterinary populations. Reduction in MR by TEER is the primary therapeutic goal because chronic regurgitation leads to progressive volume overload of the left atrium and left ventricle [26,27].

In the current study, three-dimensional echocardiography provided fine detail of valve morphology and improved device positioning during leaflet grasping. Procedural imaging after the procedure showed improved leaflet coaptation and decreased regurgitant flow, consistent with the expected hemodynamic effect of the operation. Other similar observations have been established in both human and veterinary interventional cardiology, where residual MR severity following TEER is consistently linked to clinical outcome [9,21,28]. The improvement in myocardial work parameters, including those measured by global longitudinal strain (GLS), is notable. Prior to TEER, some dogs showed significantly low GLS values and myocardial work efficiency. In chronic MR, increased preload might exaggerate myocardial deformation, leading to an apparent preservation or supranormal strain despite inefficient ventricular work. Pre-TEER GLS values became less negative, but GWI and GWE increased, which is a pattern that is more consistent with modified loading following a decrease in regurgitant volume than with impaired systolic performance. Myocardial work assessment combines strain with afterload, which is therefore more likely to offer accurate representations of real ventricular mechanics in MR-treated dogs. Pressure-strain loop analysis showed an even more homogeneous regional myocardial work distribution post-intervention.

In patients with chronic MR, myocardial deformation indices such as GLS appear preserved or exaggerated. GLS individually may fail to provide a true representation of myocardial performance following sudden changes in loading conditions. For myocardial work parameters, the afterload and ventricular pressure-strain relationships are also included, providing a more physiologically complete assessment of myocardial function. In human cardiology, several studies have demonstrated that GWI and GWE correlate with ventricular remodeling, symptom severity, and clinical outcomes following mitral valve repair or replacement. After successful TEER, improvements in myocardial work have been demonstrated, including reverse remodeling, enhanced cardiac efficiency, and improved functional status [17]. Moreover, the application of pressure-strain loop analysis has enabled detection of subtle alterations in myocardial performance that may not be detectable with ejection fraction and GLS alone.

Based on these observations, myocardial work could be particularly valuable for assessing ventricular adaptation to sudden decreases in regurgitant volume. The present study showed a significant rise in GWI and GWE after TEER and after follow-up. The enhanced myocardial work efficiency indicates that ventricular contraction was more energetically efficient following MR reduction, with an increased percentage of the work devoted to forward stroke volume rather than regurgitant flow. Regional pressure-strain loop bull’s-eye maps showed a more uniform distribution of myocardial work post-TEER, furthering the theory that TEER could partially reconstitute mechanical synchrony and ventricular efficiency. Although myocardial work has been increasingly recognized in human cardiovascular medicine, reports from veterinary cardiology have been limited. Recent studies in dogs with MMVD demonstrate that myocardial work indices capture functional adjustments directly affected by disease progression in the heart and may provide additional information beyond conventional echocardiographic markers.

This result suggests that assessment of myocardial work may provide prognostic significance. Low GWE and wasted work have been found to be negatively related to outcomes, with significant adverse effects and persistent symptoms following valve manipulation as shown in human studies [25]. Although the current study was not a long-term prognostic follow-up, the observed improvements in myocardial work indices suggest that these indicators could serve as useful clinical markers of procedural success and ventricular recovery after TEER in dogs. However, additional studies in larger-scale follow-up and longer durations are needed to determine whether myocardial work indices can predict survival, the time of congestive heart failure or recurrence of significant MR, and/or the relevance of further TEER intervention.

In addition, myocardial work analysis was performed using software originally developed and validated for human patients. Although previous veterinary studies have demonstrated its feasibility in dogs, species-specific validation remains limited. Consequently, myocardial work values should be interpreted cautiously, and further validation studies in canine populations are warranted.

A secondary clinical significance of TEER also lies in its potential to predict prognostic risk and circulating cardiac biomarkers in dogs with MMVD. Long-term MR has a role in continuous cardiac remodeling, increased neurohormonal activation, progressive heart failure, and prognosis with worsening heart failure state [29,30]. We did not further quantify biomarkers in this study; however, if MR severity decreased and myocardial work indices improved after TEER, hemodynamic adaptation would be a significant predictor of prognosis. Previous studies showed that cardiac enlargement variables (LA/Ao ratio, LV size) and pulmonary hypertension were associated with the disease prognosis in animals with MMVD and survival [31]. Reduction in MR severity and stabilization of transmitral gradients by this study may result in attenuation of persistent volume overload and ventricular remodeling. N-terminal pro-B-type natriuretic peptide (NT-proBNP), among existing biomarkers, has been extensively employed in dogs with MMVD in the assessment of myocardial wall stress and disease severity [32]. Elevated NT-proBNP levels have been linked to congestive heart failure and pulmonary hypertension and reduced survival from disease in dogs with MMVD [31,32]. After successful TEER, decreased regurgitant volume and left atrial pressure, as previously described, might theoretically reduce myocardial stretch and reduce the circulating NT-proBNP levels. The results suggest that following mitral valve repair of mitral valve dysfunction in MR patients, the results are similar to decreased concentrations of natriuretic peptides [33]. The enhanced myocardial work efficiency seen following TEER could potentially alleviate chronic myocardial stress and oxygen demand and decrease the chances of persistent injury to the myocardium after TEER.

Nevertheless, the association of TEER with further longitudinal change in cTnI concentrations in veterinary medicine is unknown and still under investigation. The myocardial work indices analyzed in the current study may indicate a prognostic effect. Moreover, improvements in GWI and GWE following TEER might reflect enhanced ventricular mechanics and increased myocardial energy utilization. In human cardiology, alterations in myocardial work parameters have been correlated with adverse cardiovascular outcomes and impairments in recovery following valvular therapy. Thus, myocardial work analysis could be a promising non-invasive tool to assess dogs with chronic ventricular dysfunction and a success-aided procedure. However, in this study, we provide preliminary clinical insight into myocardial functional adaptation following TEER in dogs with MMVD, and the study may serve as a basis for future interventional research in veterinary cardiology.

The results of this study confirm that, with a sufficient selection of cases and protocols, it is possible to achieve successful MR reduction while maintaining satisfactory transmitral flow. However, the long-term influence of increasing gradients in dogs after TEER remains to be determined. The number of dogs was small and needed long-term follow-up. Hemodynamic evaluation was performed mostly on an echocardiographic basis without invasive pressure measurements. Furthermore, the myocardial work analysis adapted in this study is based on the same methods developed in human cardiology, which have not been validated in dogs. In addition, the variations among breeds and anesthesia-associated effects may further affect myocardial work measurements. Moreover, further studies on optimal transmitral gradients, residual MR thresholds, and procedural methods may help enhance the survival and outcomes for the TEER procedure in dogs.

In the present study, among surviving Stage C and D dogs, 3/6 required reduced diuretic doses during follow-up due to improved clinical status and reduced pulmonary congestion. However, complete discontinuation of diuretic therapy was achieved in 3 dogs during the study period. Moreover, four dogs died during the follow-up period. Device-related complications included single leaflet detachment (one dog) and a presumed transient neurologic event due to suspected thrombosis (three dogs).

An important observation of the present study was the occurrence of four deaths during follow-up, three of which were associated with suspected thromboembolic complications based on acute neurological signs and clinical deterioration. Although definitive confirmation was not available because postmortem examinations were not performed, these findings suggest that thromboembolism may represent an important complication following canine TEER. During the study period, postoperative antithrombotic management consisted primarily of clopidogrel administration. Following these observations and recent reports describing device-associated thrombosis after TEER in dogs, our institutional protocol has been modified to include early postoperative low-molecular-weight heparin (enoxaparin) in combination with antiplatelet therapy. Future studies are required to determine the optimal thromboprophylaxis strategy for dogs undergoing TEER.

The deaths occurred between 1 and 4 weeks after the procedure and were not associated with intraoperative or immediate postoperative complications. Although progression of advanced MMVD likely contributed to the observed mortality, the possibility that TEER-related complications, thromboembolic events, or patient-selection factors influenced outcomes cannot be excluded. These findings highlight the importance of careful case selection and postoperative monitoring. Based on our experience, candidate selection has become more conservative, with increased consideration given to disease stage, degree of cardiac remodeling, pulmonary hypertension, systemic comorbidities, and thromboembolic risk. Consequently, the beneficial effects observed on myocardial work parameters should be interpreted cautiously until larger studies with longer follow-up and standardized thromboprophylaxis protocols become available.

Although all dogs survived the TEER procedure and immediate postoperative recovery period, four dogs died during follow-up, leading to a significant reduction in the available population for serial myocardial work assessment. As a result, survival analysis and identification of prognostic factors were beyond the scope of the present study. The observed mortality is likely attributed to the advanced nature of MMVD in some patients rather than procedural failure alone. Due to the small sample size and limited follow-up population, a definitive causal relationship between TEER, anesthesia, and mortality cannot be excluded. Thus, survival outcomes should be interpreted cautiously.

The limitations in this study include the small sample size, which likely reduced statistical power and the ability to detect associations. It was noted that 10 dogs underwent TEER in the initial assessment, whereas only 6 completed the echocardiographic and myocardial work after follow-up evaluations. The sample size for serial assessment may have affected the statistical power of the study. Therefore, the relationships among MR reduction, myocardial work indices, and cardiac remodeling should be interpreted with caution. Survivor bias was also likely due to the mortality of four dogs in follow-up. Dogs that made it to the follow-up probably included those with better hemodynamic adaptation. However, dogs with more advanced disease progression or postoperative problems may have been underrepresented in subsequent investigations. The predictors of survival or adverse outcomes were therefore limited in this study. Long-term follow-up studies should be performed with the main goal of ascertaining the prognosis for improvement in myocardial work changes and post-procedural hemodynamics in dogs undergoing a TEER intervention.

Additional limitations include the absence of comprehensive hemodynamic assessment, as cardiac output, invasive arterial blood pressure monitoring, pulmonary venous pressure, and left heart catheterization were not routinely performed. While echocardiography is an invaluable non-invasive measure of cardiac function, the invasive hemodynamic measurements remain the reference standard in assessing modulations of ventricular loading and transmitral pressure gradients following mitral valve therapy. Additionally, no systematic quality-of-life evaluations and owner-reported outcomes were obtained during follow-up. Therefore, the clinical relevance of TEER to functional status and long-term patient welfare could not be adequately assessed. Further studies including biomarkers, invasive hemodynamic monitoring, cardiac output measurements, and standardized quality-of-life questionnaires would provide a more comprehensive evaluation of treatment efficacy and prognosis.

The progression of mitral valve disease and chronic ventricular remodeling can persist for months to years after treatment, and the long-term viability of TEER in dogs can be unknown. Moreover, myocardial work analysis performed in this study was an adaptation of human cardiology software and is not yet fully standardized or validated across dog breeds and body size. Different loading requirements, heart rate, anesthesia, and image quality could have influenced strain and myocardial work parameters.

Although there are several limitations in the current study, it provides preliminary evidence of a working myocardial response to TEER in myocardial function in veterinary patients. Further study should focus on longer follow-up periods, larger sample sizes, and relationships with survival, quality of life, or progression of heart failure.

## 5. Conclusions

Transcatheter edge-to-edge repair significantly reduced mitral regurgitation in MMVD-treated dogs and improved myocardial work indices during the intervention. Global longitudinal strain decreased after TEER, whereas the global work index and myocardial work efficiency improved, indicating that volume overload correction improved ventricular mechanics. Three-dimensional echocardiography complemented analysis with 3D myocardial work data for procedural assessment and postoperative evaluation. The results affirm the feasibility of TEER in dogs. The treatment of the mitral valve and myocardial work analysis may serve a supplementary role in monitoring cardiac functional recovery after mitral valve intervention.

## Figures and Tables

**Table 1:** Baseline characteristics of the study population (N = 10 dogs).

Variable	Value
Number of dogs	10
Breed distribution: Pomeranian (N = 3), Beagle (N = 2), Maltese (N = 2), Shih Tzu (N = 1), Poodle (N = 1), Chihuahua (N = 1)	Small breeds (N = 8), Middle breed (N = 2)
Age (years)	10.7 ± 2.32
Sex:	Male (N = 5), Female (N = 5)
Body weight (kg)	8.1 ± 5.16
ACVIM stage	B2 (N = 3), C (N = 6), D (N = 1)
Heart rate (beats/min)	128 ± 18
LA/Ao ratio	1.9 ± 0.3
LVIDd (normalized)	1.85 ± 0.25
Baseline MR severity	Moderate (N = 3), Severe (N = 7)
Medication	Number of dogs (%)
Pimobendan	10 (100%)
Furosemide	8 (80%)
Spironolactone	8 (80%)
ACE inhibitors	3 (30%)
Sildenafil	4 (40%)

**Table 2:** Mitral regurgitation severity scoring system.

Parameter	Score 1	Score 2	Score 3	Score 4	Score 5	Score 6	Score 7	Score 8	Score 9	Score 10
VC (mm)	<1	1–<2	2–<3	3–<4	4–<5	5–<6	6–<7	7–<8	8–<10	≥10
MRV (mL/beat)	<1	1–<2	2–<3	3–<4	4–<5	5–<6	6–<8	8–<10	10–<12	≥12

**Table 3:** Echocardiographic and myocardial work parameters before (N = 10), and immediately after transcatheter edge-to-edge repair (TEER) in dogs with MMVD (N = 10).

Parameter	Pre-TEER	Immediately Post TEER	*p*-Value
Mitral regurgitation severity score	12.2 ± 4.4	7.1 ± 3.8	<0.0005
Vena contracta (mm)	5.9 ± 1.5	3.8 ± 1.6	<0.0001
Mitral regurgitant volume (mL/beat)	8.4 ± 6.4	3.3 ± 4.1	0.0058
LA/Ao ratio	2.2 ± 0.6	1.9 ± 0.3	0.012
LVIDdN	1.9 ± 0.3	1.76 ± 0.3	0.039
Fractional shortening (%)	34.7 ± 8.6	33.3 ± 11.9	0.5855
Transmitral gradient (mmHg)	3.03 ± 0.65	1.01 ± 0.48	<0.005
Global longitudinal strain (%)	−22.1 ± 4.8	−27.8 ± 2.9	0.005
Global work index (mmHg%)	921.7 ± 168	1149.3 ± 174	0.0847
Global constructive work (mmHg%)	2238 ± 560	2311 ± 314	0.1955
Global wasted work (mmHg%)	338.3 ± 188	179 ± 64.7	<0.05
Global work efficiency (%)	84.9 ± 3.6	92.7 ± 2.6	<0.05

## Data Availability

The original contributions presented in this study are included in the article. Further inquiries can be directed to the corresponding authors.

## Abbreviations

The following abbreviations are used in this manuscript:

MMVD	Myxomatous mitral valve disease
MRV	Mitral valve regurgitant volume
GLS	Global longitudinal strain
GWI	Total myocardial work index during systole
GCW	Myocardial work contributing to LV ejection
GWW	Myocardial ineffective work that contributes to no forward displacement
GWE	Ratio of constructive work to total work
TEER	Transcatheter edge-to-edge repair
VC	Vena contracta
